# Satisfaction with Health Care Services in the Adult Population of the Federation of Bosnia and Herzegovina during the COVID-19 Pandemic

**DOI:** 10.3390/medicina59010097

**Published:** 2022-12-31

**Authors:** Šeila Cilović-Lagarija, Sanjin Musa, Stela Stojisavljević, Nino Hasanica, Elma Kuduzović, Mirza Palo, Marek Majdan, Martha Scherzer, Katrine Bach Habersaat, Catherine Smallwood, Ardita Tahirukaj, Dorit Nitzan

**Affiliations:** 1 Department of Statistic, Institute for Public Health FB&H, 71000 Sarajevo, Bosnia and Herzegovina; 2 Department of Epidemiology, Institute for Public Health FB&H, 71000 Sarajevo, Bosnia and Herzegovina; 3 Department of Social Medicine, Public Health Institute of the Republic of Srpska, 78000 Banja Luka, Bosnia and Herzegovina; 4 Medical Faculty, University of Banja Luka, 78000 Banja Luka, Bosnia and Herzegovina; 5 Institute for Health and Food Safety Zenica, Institute for Public Health, 72000 Zenica, Bosnia and Herzegovina; 6 Country Office in Bosnia and Herzegovina, World Health Organization, 71000 Sarajevo, Bosnia and Herzegovina; 7 Health Emergencies Programme, World Health Organization Regional Office for Europe, 2100 Copenhagen, Denmark; 8 Institute for Global Health and Epidemiology, Faculty of Health Sciences and Social Work, Trnava University, Hornopotocna 23, 91843 Trnava, Slovakia; 9 Country Health Programmes, World Health Organization Regional Office for Europe, 2100 Copenhagen, Denmark

**Keywords:** satisfaction, health care, COVID-19, federation of Bosnia and Herzegovina, EUROPEP

## Abstract

*Background and Objectives*: Patient satisfaction with health care can influence health care-seeking behavior in relation to both minor or major health problems or influence communication and compliance with medical advice, which is especially important in emergencies such as the COVID-19 pandemic. Thus, it is important to continually monitor patient satisfaction with provided care and their dynamics. The aim of this study was to assess patient satisfaction with health care during the COVID-19 pandemic in the adult population of the Federation of Bosnia and Herzegovina (FB&H) and compare it with levels of satisfaction in the same population before the COVID-19 pandemic. *Materials and Methods*: A representative, population-based survey was implemented in the adult population of the FB&H using the EUROPEP instrument, which measures satisfaction with health care using 23 items. The sample included 740 respondents who were 18 years or older residing in the FB&H and was implemented in December 2020. All data were collected using a system of online panels. The survey questions targeted the nine months from the beginning of the pandemic to the time of data collection, i.e., the period of March to December 2020. *Results*: The mean composite satisfaction score across all 23 items of the EUROPEP tool was 3.2 points in all age groups; the ceiling effect was 22% for the youngest respondents (18–34 years old), 23% for 35–54 years old, and 26% for the oldest group (55+), showing increasing satisfaction by age. The overall composite score for both females and males was 3.2. The ceiling effect was higher in those with chronic disease (29% vs. 23% in those without chronic disease). The composite mean score for respondents residing in rural vs. urban areas was 3.2 with a ceiling effect of 22% in rural and 24% in urban residents. When comparing mean composite scores surveyed at various points in time in the FB&H, it was found that the score increased from 3.3 to 3.5 between 2011 and 2017 and dropped again to 3.3 in this study. Despite these observations in the overall trends of satisfaction scores, we note that no statistically significant differences were observed between most of the single-item scores in the stratified analysis, pointing to the relative uniformity of satisfaction among the analyzed population subgroups. *Conclusions:* The rate of satisfaction with health care services in the FB&H was lower during the COVID-19 pandemic compared to 2011 and 2017. Furthermore, while an increasing trend in satisfaction with health care was observed in the FB&H during the years prior to 2020, the COVID-19 pandemic may have contributed to the reversal of this trend. It is important to further monitor the dynamics of patient satisfaction with health care, which could serve as a basis for planning, delivering, and maintaining quality services during the COVID-19 pandemic and other emergencies.

## 1. Introduction

With over 265 million confirmed cases and over 2.1 million confirmed deaths by December of 2022, the COVID-19 pandemic poses a major public health, medical, and societal challenge throughout the WHO European region [1]. Of those patients who do become symptomatic with COVID-19, most people develop only mild or moderate disease, with about 15% requiring oxygen support and 5% developing critical disease with complications [2]. While these proportions may seem relatively low, with large numbers of new cases, they may present a substantial burden on health systems. In addition, the disease has been associated with a post-COVID condition characterized by symptoms persisting long-term [2], which puts additional strain on health care providers and further limits access to essential health services (EHS) [3].

Indeed, major disruptions in EHS were observed globally, as documented by the second round of the WHO National Pulse Survey on continuity of EHS during the COVID-19 pandemic: Of the 135 responding countries, 94% reported at least one service disruption, and disruptions were reported across all health areas, demonstrating the far-reaching impact of the pandemic on health systems. In 66% of countries, health workforce-related disruptions represented the most common causes of service disruptions on the supply side [4].

The rapid spread of the virus has caused major changes in the functioning of health systems, which resulted in a need for decisions to reorganize the health workforce and health care provision (e.g., reorganization of work schedules and hours, changes in location and working conditions, use of equipment, etc.). In the Federation of Bosnia and Herzegovina (FB&H), the COVID-19 pandemic also revealed the lack of health professionals in clinical disciplines (such as family medicine, pulmonology, anesthesiology, and resuscitation) and public health disciplines, especially in the field of epidemiology [5]. In general, these circumstances and the increased work stress put a tremendous toll on the mental health of health workers [6]. While it is important to monitor the disruptions on the level of health care providers, it is equally important to gain insights into these disruptions from the perspective of the patient, including their perceived satisfaction with health care, i.e., the degree to which patients are pleased with their health care both inside and outside health care facilities [7,8].

Previous studies showed that patient satisfaction with health care can influence health care-seeking behavior in relation to both minor or major health problems or influence communication and compliance with medical advice [7,9,10,11]. Satisfied patients were shown to be more likely to comply with treatment, which is especially important in emergencies such as the COVID-19 pandemic where reductions in procedure and treatment adherence, increased treatment dissatisfaction, and discontinuance of patient treatment follow-up were observed [12]. Furthermore, patient satisfaction and patient evaluation of health care reflect the level to which the patient’s subjective and objective needs have been met while the surveying itself gives patients the impression that their opinion is valued [13]. Thus, it is important to continually monitor patient satisfaction with provided health care and their dynamics.

Several tools have been designed to assess patient satisfaction with health care: A recent systematic review evaluated 13 such tools [14] and concluded that the EUROPEP tool [15] has been the most internationally validated of them and thus is suitable for use in international settings. In addition, the tool has been successfully used to compare patient satisfaction with health care during the pre- and post-economic crisis periods in Greece, which documents its sensitivity in showing the dynamics of satisfaction in that time and creates grounds for its application to compare patient satisfaction with care in the pre-COVID-19 period and during the COVID-19 pandemic [16]. The countries where the tool has been previously used include BiH, where it was applied in the study of patient satisfaction with primary health care in families and general medicine in Zenica [17], and thus the tool has been validated for the country’s population and the obtained satisfaction scores are available to be used as a baseline to assess the impact of the COVID-19 pandemic on patient satisfaction with health care.

Bosnia and Herzegovina is divided into two entities: the Federation of Bosnia and Herzegovina (FB&H) and the Republic of Srpska (RS). Besides these, the Brčko District is part of the federation. The FB&H is further divided into 10 cantons, has a population of 2.2 million, and a GDP per capita of 7782 USD [18]. The health care system in the FB&H is organized on federal, cantonal, and municipality levels with different jurisdictions determined by law. The organization of the health care system on a cantonal level with coordination from the federal level presents an opportunity for decentralization of the health care system following the examples of countries with well-established health systems. In the FB&H, primary health care is provided through 80 primary health centers, and the secondary and tertiary levels of health care are provided through 24 clinical centers and hospitals. There are 11 regional institutes of public health [18]. The FB&H faces the aging of its population, which comes along with the increasing prevalence of chronic noncommunicable diseases and risk factors for these diseases, which, on one hand, had an influence on the impact of the COVID-19 pandemic on the country’s population, and, on the other hand, warrants for action to increase the quality of health services [19].

Patient satisfaction with health care brings important insight into the quality of provided health care, as it reflects the level to which patient needs have been met. Better information on the factors affecting satisfaction will guide health care providers and planners to improve the quality of the service they deliver to users and will ensure that reliable information can be collected for the decision-making process. The COVID-19 pandemic has placed additional strain on health care providers and further limited access to essential health services (EHS). The aim of this study was to assess patient satisfaction with health care during the COVID-19 pandemic in the adult population of the FB&H using the EUROPEP questionnaire and compare it with levels of satisfaction in the same population before the COVID-19 pandemic.

## 2. Materials and Methods

### 2.1. Study Design, Population, and Data Collection

Several tools have been designed to assess patient satisfaction with health care: A recent systematic review evaluated 13 such tools [16] and concluded that the EUROPEP tool [17] has been the most internationally validated and thus suitable for use in international settings. In addition, the tool has been successfully used to compare patient satisfaction with health care in the pre- and post-economic crisis period in Greece, which documents its sensitivity in showing dynamics of satisfaction in that time and creates grounds for its application to compare patient satisfaction with health care in the pre-COVID-19 period and during the COVID-19 pandemic [18]. The tool was previously used in BiH to assess patient satisfaction with primary health care in Zenica [19], and the obtained satisfaction scores are available to be used as a baseline to assess the impact of the COVID-19 pandemic on patient satisfaction with health care.

A representative, population-based survey was implemented in the adult population of the FB&H using the EUROPEP instrument [17]. The EUROPEP instrument is a 23-item validated and internationally standardized measure of patient evaluations of general practice health care developed in the years 1995–1998 and further revised in 2006; it has been used in about 20 countries, including the FB&H [17]. For the purposes of this study the EUROPEP tool along with the WHO Europe’s population survey on the impact of the COVID-19 pandemic on disruptions, access and patterns of use of EHS, health-seeking behaviors, and population health and wellbeing from the community perspective were integrated into the WHO Behavioral Insights survey [20]—all three surveys were rolled out as one. This paper only reports on the findings from the EUROPEP tool items.

The satisfaction with health care using the EUROPEP tool was measured for each of the 23 items on a 5-point scale, 5 being the highest possible score. The instrument was translated into the Bosnian language and applied to a representative sample of 740 respondents 18 years or older residing in the FB&H in December 2020. All data were collected by a professional survey research company using online panels, with data collection and data delivery conducted within 72 h from survey initiation. Sampling, quota monitoring, and invitational activities were performed using appropriate methodology to achieve representativeness of FB&H sample in terms of age, sex, and geographical distribution as described in the protocol of WHO’s Behavioral Insights survey in FB&H [21]. The survey questions targeted the nine months from the beginning of the pandemic to the time of data collection, i.e., the period of March to December 2020.

### 2.2. Data Analysis

The sample of respondents was stratified by age (18–34 years old, 35–54 years old, and 55 years and older), gender (males, females), residence (urban vs. rural), and by presence or absence of at least one chronic noncommunicable disease (e.g., those who at the time of survey had at least one chronic noncommunicable disease—such as diabetes, hypertension, chronic cardiovascular disease, or neoplasms vs. those who had no such disease). First, mean satisfaction scores with 95% confidence intervals (CI) were calculated for each of the 23 items of the instrument for the whole sample of respondents and each stratum. Furthermore, ceiling effects [22] were calculated for each of the items, which represent the proportion of respondents choosing the highest possible assessment on the provided scale (e.g., “5”); these were also calculated for the whole sample and each stratum. All statistical analyses were performed using the R statistical language. *t*-test and one-way ANOVA tests were used to compare the means between the strata. *p*-values were calculated and considered statistically significant if below 0.05.

### 2.3. Ethical Considerations

As an observational study with voluntary participation of the general population, expected risk for participants was considered low. National ethical approval from Ethical Broad Institute of Public Health of FB&H as well as approval from the WHO Ethical Review Committee was obtained before the start of data collection. Before completing the online questionnaire, participants were duly informed and asked for their consent. Participation was voluntary; they could withdraw from participation at any time, and nonparticipation would not have any negative effects. Throughout the survey, the ICC/ESOMAR International Code on Market and Social Research [23] was observed and adhered to.

## 3. Results

### 3.1. Demographic Characteristics

In total, 740 respondents participated in the survey. The mean age was 44 years, and the youngest respondent was 18 years old; the oldest was 73 years old. A total of 233 respondents were in the 18–34 years age group (32%), 327 were in the 35–54 years age group (44%), and 180 (24%) were in the 55 or older group. The gender distribution was 51% female and 49% male. Most of the respondents lived in an urban area (471, 64%). Of the 740 respondents in total, 664 provided information on the presence or absence of chronic noncommunicable disease—of these, 170 (26%) reported that they had at least one chronic noncommunicable disease.

### 3.2. Satisfaction with Health Care by Age

The mean composite satisfaction score (e.g., the mean score across all 23 items surveyed) was 3.2 (95% CI: 3.1–3.3) points in all three age groups. This was not different from the composite score of the total sample at 3.2 (3.2–3.3). A statistically significant difference between the scores was observed for two items: The score was 3.7 (95% CI: 3.6–3.9) points significantly higher in 18–34-year-olds compared with the 55+ group for the item “Does the doctor perform a physical examination?” (*p* < 0.01), and the satisfaction score in the youngest age group was significantly lower for the item “Do you feel better when you tell the doctor about your problem?” (*p* = 0.049) compared to respondents 55 years or older. The ceiling effect was 22% for the youngest respondents, 23% for 35–54-year-olds, and 26% for the oldest group, showing increasing satisfaction by age. See Table 1 for details.

### 3.3. Satisfaction with Health Care by Gender

The overall composite score for females was 3.2 (95% CI: 3.1–3.3) and 3.2 (95% CI: 3.1–3.4) for males. There is no significant difference between the composite scores for females or males or with composite score for the whole sample of 3.2 (95% CI: 3.2–3.3). There were no statistically significant differences in the mean scores between the gender groups across the items except for the item “Do you feel better when you tell the doctor about your problem?” where the score of 3.5 (95% CI: 3.4–3.7) was significantly higher in females than males (*p* = 0.04). The ceiling effect was 24% overall in females (ranging from 17% to 33%) and 23% in males (ranging from 17% to 32%) with no significant differences. See Table 2 for details.

### 3.4. Satisfaction with Health Care by Presence of Chronic Noncommunicable Disease

Differences between the two items were statistically significant when respondents with and without chronic diseases were compared: Satisfaction was higher in those with chronic disease for the item “Do you feel better when you tell the doctor about your problem?”—mean scores of 3.7 (95% CI: 3.5–3.9) vs. 3.4 (95% CI: 3.2–3.5), *p* < 0.01—and those without a chronic disease assigned a higher score to the item “Does the doctor perform a physical examination?” compared to those with a chronic disease—mean scores 3.6 (95% CI: 3.5–3.7) vs. 3.3 (95% CI: 3.1–3.6), *p* = 0.028. The ceiling effect was higher in those with chronic disease (29% vs. 23% in those without chronic disease). See Table 3 for details.

### 3.5. Satisfaction with Health Care by Residence

The composite mean score for respondents residing in rural areas was 3.2 (95% CI: 3.1–3.4) and 3.2 (95% CI: 3.1–3.3) for those living in urban areas. The ceiling effect was 22% in rural and 24% in urban residents. There were no statistically significant differences between the groups across the 23 items—the mean scores ranged from 2.9 to 3.6 in both the rural and urban groups. Details are given in Table 4.

### 3.6. Comparison of the Dynamics from 2011 to 2020

**Figure 1:**
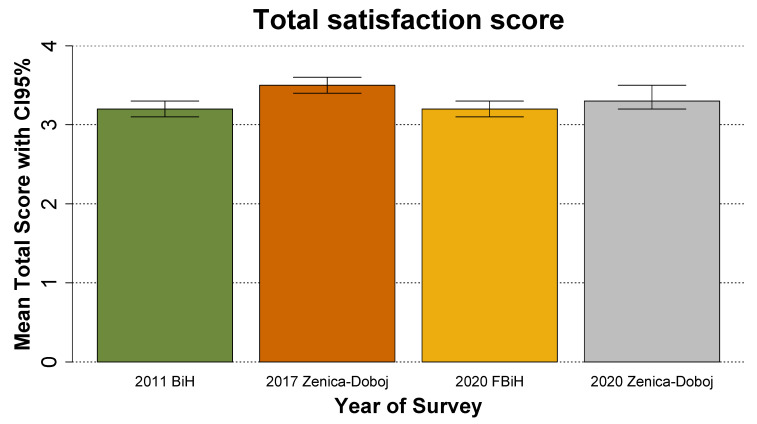
Satisfaction with health care in the adult population of the Federation of Bosnia and Herzegovina and the Zenica-Doboj region in 2011, 2017, and during the COVID-19 pandemic. Source of data for 2011 and 2017 [13].

Figure 1 presents a comparison of four composite mean scores of patient satisfaction that come from three different surveys implemented in the FB&H over a period of 10 years. The mean score based on the survey in 2011 was estimated at 3.2 (95% CI: 3.1–3.3). A repeated survey before the COVID-19 pandemic that was implemented only in the Zenica-Doboj canton of the FB&H in 2017 showed an increase in the composite satisfaction score to 3.5 points (95% CI: 3.4–3.6). The survey from 2020 reported here showed a decrease in a level of satisfaction that is similar to the one measured in 2011—a mean composite score of 3.3 (95% CI: 3.2–3.5). For the purposes of a better comparison of scores between 2017 and 2020, Figure 1 also shows the mean score for 2020 only for the Zenica-Doboj region—the comparison of this score to the score from the same region from 2017 also shows a decrease in the overall level of satisfaction with health care in 2020 during the COVID-19 pandemic as compared to the period before the pandemic (2017).

## 4. Discussion

### 4.1. Main Findings

A cross-sectional, population-based study on a representative sample of 740 respondents 18 years or older in the FB&H was conducted to assess patient satisfaction with health care during the COVID-19 pandemic. The mean composite satisfaction score across all 23 items of the EUROPEP tool was 3.2 points in all age groups; the ceiling effect was 22% for the youngest respondents (18–34 years old), 23% for 35–54-year-olds, and 26% for the oldest group (55+), showing increasing satisfaction by age. The overall composite score for both females and males was 3.2. The ceiling effect was higher in those with chronic disease (29% vs. 23% in those without chronic disease). The composite mean score for respondents residing in rural vs. urban areas was 3.2 with the ceiling effect being 22% in rural and 24% in urban residents. When comparing mean composite scores surveyed at various points in time in the FB&H, we found that the score increased from 3.3 to 3.5 between 2011 and 2017 and dropped again to 3.3 in this study, suggesting a decrease in satisfaction with health care in 2020 during the COVID-19 pandemic (compared to 2017). Despite these observations in the overall trends of satisfaction scores, we note that no statistically significant differences were observed between the single-item scores in the stratified analysis, pointing to the relative uniformity of satisfaction among the analyzed population subgroups.

### 4.2. Interpretation of Results and Comparison with Published Literature

Several studies reported previously on levels of patient satisfaction in the FB&H, which provides a reference to observe its dynamics over time. In the study of patient satisfaction with health care in the Zenica-Doboj Canton using the EUROPEP questionnaire, a statistically significant difference was observed between mean patient satisfaction in 2011 and mean patient satisfaction in 2017—in favor of 2017 [13]. In another study conducted in the Zenica-Doboj Canton, differences in patient satisfaction in favor of family medicine were statistically most significant when it came to scheduling examinations at times convenient to the patient, the possibility of telephone links with the office, and long waiting times in the waiting room. The collected data confirm the high level of patient satisfaction with the family medicine units of primary health care [17]. While these studies were conducted only in the population of the Zenica-Doboj Canton, their generalizability to the whole FB&H may be limited. However, when the findings of the study presented here are compared between the whole FB&H excluding the Zenica-Doboj region and between the Zenica-Doboj Canton only, no significant differences are observed (Figure 1, Table 5). Further limitations to such comparisons may be introduced by differences in the demographics of the samples used in the studies; however, previous studies into the topic in the FB&H report similar age and gender distributions to those seen in this study, which suggest no substantial role of such bias [7,13,17,24,25]. Furthermore, all listed studies have used the same survey tool to collect the data. Thus, it can be assumed that the findings of this study can be compared to the findings of previous studies.

This study compared scores of patient satisfaction between the period before COVID-19 and during the pandemic. A similar study was conducted in Saudi Arabia, aiming to evaluate the impact of the COVID-19 pandemic on patient satisfaction with health care, which showed that most patients reported very high scores for nurses and physicians in most of the surveyed items [26]. Another study showed that with the COVID-19 pandemic, patients in all health care facilities had high expectations regarding the quality of services and were satisfied with the overall service provided by pharmacists with a grade of 4.53 [27]. In another study, the overall satisfaction with medical services delivered by family physicians was 80%, and continuity and confidentiality constituted the higher satisfaction rate at 97% while informativeness satisfaction constituted 90%; accessibility and acceptability had the lowest satisfaction rate [28]. A survey in the US revealed that patient satisfaction fell 13% during the pandemic when compared to previous periods [29].

While uniformity in the observed scores has been observed in most of the items between the analyzed sample strata (e.g., by age, gender, presence of chronic disease, or residence), in some specific items, statistically significant differences were found: Speaking to the physician about the health problem gained higher scores among older patients (55 years or older) compared to younger ages, in females compared to males, and in respondents with chronic noncommunicable diseases compared to those without such a disease. This suggests that spending time with the patient and discussing their health problems is very important, especially among the most vulnerable population groups (e.g., the elderly and those chronically ill). While it is difficult to maintain sufficient direct contact, a wide application of tools such as telemedicine may provide an alternative way of contact to overcome these problems, as shown by previous studies focusing on health care provision during the COVID-19 pandemic. Multiple studies reported that health care providers responded by rapidly transitioning from in-person to video consultations, which translated to a substantial increase in health care uptake via these tools [30,31]. Higher satisfaction rates were observed in older and male patients [32].

We note that despite the progress in patient satisfaction with health care made between 2011–2017 in the FB&H, the observed scores lag behind countries with stronger economic levels and better-organized health systems: An assessment using the same tool performed in eight European countries (Austria, Belgium, France, Germany, the Netherlands, Slovenia, Switzerland, and the UK) yielded overall proportions of respondents assigning the two highest scores (4 or 5) ranging from 83% to 93% [33]; a Dutch study estimated such proportions at 58–91% among the 23 items [34]; and a study from Italy reported this proportion on average at 80% [35].

While in our study we report ceiling effects (e.g., proportions of those assigning the highest possible scores), when recalculated for the purposes of comparison with these studies, we found that overall, 45% of respondents in our study assigned the two highest possible scores. The average ceiling effect for all 23 items in our study was 25% while those reported from other countries were higher: A study from Norway yielded a ceiling effect score of up to 80% [36] while a study from Bulgaria estimated ceiling effects ranging between 34–68% [37]. Thus, satisfaction appears to be worse compared to some other European countries.

The ceiling effect reported in our study is relatively low compared to other studies using the EUROPEP tool [34,35]. This suggests that the responses in this study were more evenly distributed among the five response categories, which increases the usability of mean scores as the main measure of satisfaction in the respective domains (in case the ceiling effect is very high, using means is not suggested, as it may be skewed by the uneven distribution among the responses within the response categories) and supports the validity of the EUROPEP tool to capture satisfaction with health care in the population of the FB&H [22].

Furthermore, in a survey of the consequences of COVID-19 on society in BiH conducted by UNICEF and UNDP using the CATI method, about 12% of respondents stated that their healthcare needs were not met and that they could not be treated or receive therapy for other diseases than COVID-19 during the pandemic, particularly among vulnerable groups [38].

It may be argued that some of the observed decreases in satisfaction with health care may be attributed to changes occurring to the health care system in the FB&H between 2017 and 2020. However, in this regard, we note that based on the Reports on Health Status and Organization of Health Care in the FB&H for the years 2017, 2018, 2019, and 2020, there were no major changes to the organization of health care.

The number of health workers in the FB&H did not change significantly during the period between 2017–2020 (rate of 1.20/100,000 inhabitants in 2017; 1.22 in 2018; 1.21 in 2019). In total, there were 27,517 employees in the public health care sector in 2020, which is slightly more than in 2019 (26,811 employees) [37,38,39,40]. During this period, slightly more than ¼ of the medical doctors were 55 years old or older (29% in 2017; 30% in 2018; and 29% in 2019) [37,38,39]. A significant proportion of medical doctors over the age of 55 was especially apparent among family medicine specialists (33% in 2017; 42% in 2018) [37,38,39]. According to the age structure profile of employees in the public health care sector in the FB&H in 2020, the majority of medical doctors were older than 55 (28%) while among pharmacists, more than half were in the age group up below 44 years (57%).

Although the reform of the health care system in the FB&H is centered around strengthening primary health care (PHC), there are differences in the availability of health care to the population by cantons of the FB&H. Contrary to strategic commitments, according to which about 60% of all requests for health care should be met at the level of PHC, there has been an increase in referrals to specialists. According to health statistics data for 2020, PHC services in the FB&H (family medicine, health care of preschool and school-age children, emergency medical care, women’s reproductive health care, community mental health centers, polyvalent community nurses, occupational medicine, etc.) employed 1891 medical doctors (35% of the total number of medical doctors) and 3289 nurses/technicians (25% of all such employees), i.e., 87 medical doctors and 151 nurses/technicians per 100,000 population. In 2019, 1771 medical doctors and 3416 nurses/technicians were employed at the PHC level [40].

In 2020, the total number of visits to medical doctors in family medicine services in the public health sector was 6,346,521, i.e., 14% less than in 2019 (7,243,588), which was affected by the COVID-19 pandemic and change in the organization of work in health care centers in all cantons. The number of visits per medical doctor averaged 5.9 per year or 26 per day, which is less than in 2019 (the average number of visits per medical doctor per year was 7.1 and 31 per day) [40].

It is therefore unlikely that the decline in patient satisfaction between 2017 and 2020 could have been caused by such factors. On the other hand, at the beginning of the pandemic in January 2020, the government of the FB&H ordered restrictive measures and recommendations from the Crisis Headquarters of the Federal Ministry of Health [41]. All nonemergency health services in the FB&H have been suspended until further notice (including vaccination, preventive examinations, nonemergency surgery, etc.). This supports our conclusions that the disruptions of EHS caused by the pandemic may have led to the observed decrease in patient satisfaction with care.

Such findings warrant action towards improving health care provision, maintaining EHS, and improving satisfaction with health care among patients. The WHO developed an operational guidance for maintaining essential health services for the COVID-19 context and suggests operational strategies on how to adjust governance, prioritize EHS, optimize service delivery, establish safe and effectiepatient flow, rapidly optimize health workforce, maintain the availability of medications, equipment and supplies, fund public health and remove barriers to access, strengthen communication and monitoring of EHS, and use digital platforms to support EHS delivery [3]. Focusing on these strategies may improve the delivery of EHS while maintaining patient satisfaction with health care. All these measures were incorporated into the Crisis Preparedness and Response Plan for the Emergence of the New Coronavirus (COVID-19) in the FB&H [42]. Nevertheless, the pandemic has placed unprecedented demands on individuals and informal caregivers (including families, friends, and neighbors) in the FB&H to self-manage many health needs.

A key issue to address to improve patient satisfaction is to optimize the health workforce. To support and guide countries in such efforts, the WHO developed guidance with recommendations at the individual, management, organizational, and system levels to improve human resource management during the COVID-19 pandemic (including support and protection for health workers, strengthening health workforce teams, increasing capacity, and strengthening of health system human resources) [39]. During the pandemic of COVID-19, medical staff has been exposed to additional stressors. The data confirm the importance of the timely development of strategies for the prevention, treatment, and rehabilitation of persons with burnout, which would maintain the mental health of medical staff but also the quality of health care provided to patients within the health system [40,41].

The main goal of health care providers on all levels is to provide high-quality health care to patients and meet their needs and expectations even during a health emergency, such as the COVID-19 pandemic. Satisfaction with health care is, therefore, one of the most important determinants of evaluation of service quality by patients or their families and helps to align the provision of health care with patient expectations as an important step in defining and delivering high-quality services [42]. The quality of health care services and patient satisfaction have both been affected by the COVID-19 pandemic [43] and should be viewed as a reflection of how well patients’ subjective and objective needs are met [13]. For example, in Slovenia, no significant differences were registered in satisfaction with health care during the COVID-19 pandemic and pre-pandemic periods. Patients were statistically significantly more satisfied with information on doctors’ and health care professionals’ availabilities and respect for privacy during the pandemic Research also showed that there may be a relationship between trust in the new safety protocols established in health centers, medical service quality, perceived value according to EHC protocol, and user satisfaction in this new health care setting. The operational guidance for maintaining EHS for the COVID-19 context underlines the importance of implementing similar measures aimed at improving patients’ satisfaction with health care and access to EHS.

### 4.3. Limitations

The study relies on data from a population-based online survey. The findings reflect self-perceived and self-reported characteristics which may result in reporting bias. Using online web panels may produce bias against people without access to an internet connection, computers, smartphones, and other digital devices, including potentially the elderly population and disadvantaged population groups. However, the panels used included people in all age groups, and a concerted effort was made to ensure the inclusion of the elderly age group. We note that all efforts were made to overcome these biases by applying a valid and sophisticated sampling strategy in all steps of the implementation of the survey and analysis of its findings. Thus, despite these limitations, the study presents very valuable findings that describe the views and perceptions of satisfaction with health care by the adult population of the FB&H during a period of the COVID-19 health emergency. Furthermore, although patient satisfaction surveys are common, the answers given by patients to these questions are subjective and their interpretation can be very complex. Limitations of patient satisfaction research include the fact that they sometimes do not deal with “dissatisfaction” but simply assess the level of “satisfaction”. Responses may further be influenced by socially desirable attitudes or other biases, patient demographics, gratitude, or self-protection. Lastly, satisfaction may not necessarily reflect whether appropriate clinical action was performed; therefore, any further interpretations about health services or patient interactions with health services are limited. Considering only the proportions reporting the highest scores are considered here, actual patient satisfaction levels might be lower than those estimated.

## 5. Conclusions

The rate of satisfaction with health care services in the FB&H was lower during the COVID-19 pandemic compared to 2011 and 2017. Furthermore, while an increasing trend in satisfaction with health care was observed in the FB&H during the years prior to 2020, the COVID-19 pandemic may have contributed to the reversal of this trend. It is important to further monitor the dynamics of patient satisfaction with health care, which could serve as a basis for planning, delivering, and maintaining quality services during the COVID-19 pandemic and other emergencies.

## Figures and Tables

**Table 1:** Satisfaction with health care in the adult population of the Federation of Bosnia and Herzegovina during the COVID-19 pandemic by age group [13].

Question	Age Group (Years)								*p*-Value
	18–34		35–54		55+		Total		
	*Mean (CI 95%)*	*CE (%)*	*Mean (CI 95%)*	*CE (%)*	*Mean (CI 95%)*	*CE (%)*	*Mean (CI 95%)*	*CE (%)*	
Do you think that the family doctor spends sufficient time with you?	2.8 (2.6–3)	15%	3 (2.8–3.1)	19%	3.1 (2.9–3.3)	24%	2.9 (2.8–3)	19%	0.161
Does the doctor show any interest in your problem?	3.1 (2.9–3.2)	18%	3.2 (3.1–3.4)	24%	3.2 (3–3.4)	27%	3.2 (3.1–3.3)	22%	0.392
Do you feel better when you tell the doctor about your problem?	3.2 (3.1–3.4)	23%	3.4 (3.3–3.6)	30%	3.6 (3.4–3.8)	37%	3.4 (3.3–3.5)	30%	0.049
Does the doctor involve you in making a decision about your treatment?	3.3 (3.1–3.5)	22%	3.3 (3.2–3.5)	23%	3.4 (3.2–3.6)	30%	3.3 (3.2–3.4)	25%	0.753
Does the doctor listen to you carefully while you are presenting your problems?	3.3 (3.1–3.5)	25%	3.4 (3.2–3.6)	25%	3.4 (3.1–3.6)	32%	3.3 (3.2–3.4)	27%	0.763
Does the doctor provide you with all information about your diseases?	3.2 (3.1–3.4)	23%	3.3 (3.2–3.5)	24%	3.4 (3.2–3.6)	28%	3.3 (3.2–3.4)	25%	0.587
Does the doctor try hard to relieve your symptoms as soon as possible?	3.4 (3.2–3.6)	27%	3.4 (3.2–3.5)	23%	3.4 (3.2–3.6)	31%	3.4 (3.3–3.5)	26%	0.907
Does the doctor help you feel better and return to your everyday work?	3.3 (3.1–3.4)	21%	3.4 (3.3–3.5)	24%	3.4 (3.2–3.6)	29%	3.4 (3.2–3.5)	24%	0.371
Does the doctor perform a physical examination?	3.7 (3.6–3.9)	33%	3.4 (3.3–3.6)	26%	3.4 (3.2–3.6)	30%	3.5 (3.4–3.6)	29%	<0.01
Does the doctor perform a detailed physical examination?	3.2 (3.1–3.4)	18%	3.2 (3.1 3.4)	23%	3.2 (3–3.4)	24%	3.2 (3.1–3.3)	22%	0.946
Does the doctor work on the prevention of various diseases?	3 (2.8–3.2)	18%	2.9 (2.7–3.1)	18%	2.8 (2.6–3.1)	16%	2.9 (2.8–3)	17%	0.513
Does the doctor explain why you need to undergo additional tests and analyses?	3.2 (3.1–3.4)	22%	3.2 (3.1–3.4)	23%	3.3 (3.1–3.5)	26%	3.3 (3.2–3.3)	23%	0.975
Does the doctor provide an explanation about your symptoms and disease?	3.3 (3.1–3.4)	22%	3.3 (3.1–3.4)	24%	3.3 (3.1–3.5)	26%	3.3 (3.2–3.4)	24%	0.993
Does the doctor help with your emotional problems related to your health condition?	2.8 (2.6–3)	16%	3 (2.8–3.1)	18%	3 (3.8–3.2)	19%	2.9 (2.8–3)	18%	0.293
Does the doctor explain to you why it is important to comply with his/her instructions?	3.3 (3.1–3.5)	21%	3.2 (3–3.3)	20%	3.3 (3–3.5)	25%	3.2 (3.1–3.3)	22%	0.51
Does the doctor explain what he/she is doing during the examination?	3.3 (3.1–3.5)	22%	3.2 (3–3.3)	23%	3.1 (2.9–3.3)	22%	3.2 (3.1–3.3)	22%	0.328
Does the doctor explain what you can expect at a specialist examination in-hospital?	3 (2.9–3.2)	17%	3 (2.8–3.1)	18%	2.9 (2.7–3.1)	16%	3 (2.9–3.1)	17%	0.656
Are you assisted by other medical staff (nurse at the clinic)?	3.3 (3.2–3.5)	20%	3.4 (3.3–3.5)	21%	3.3 (3.1–3.5)	24%	3.4 (3.3–3.4)	21%	0.726
Can you make an appointment with the doctor?	3.5 (3.4–3.7)	28%	3.6 (3.5–3.7)	32%	3.5 (3.3–3.7)	35%	3.6 (3.5–3.7)	32%	0.811
Is it easy to make a phone call to the doctor?	3 (2.8–3.2)	19%	2.8 (2.7–3)	20%	2.9 (2.6–3.1)	22%	2.9 (2.8–3)	20%	0.37
Can you seek advice from the doctor by phone?	3 (2.8–3.2)	21%	2.9 (2.7–3)	20%	2.9 (2.7–3.2)	22%	3 (2.8–3)	21%	0.497
Do you wait long in the waiting room?	3.4 (3.2–3.5)	25%	3.3 (3.2–3.4)	24%	3.3 (3–3.5)	27%	3.3 (3.2–3.4)	25%	0.677
Does the doctor respond fast in an emergency situation?	3.4 (3.2–3.5)	26%	3.5 (3.4–3.6)	29%	3.6 (3.4–3.8)	32%	3.5 (3.4–3.6)	29%	0.225
Total mean score	3.2 (3.1–3.4)	22%	3.2 (3.1–3.3)	23%	3.2 (3.1–3.4)	26%	3.2 (3.2–3.3)	23%	0.946

CI: confidence interval; CE: ceiling effect (refers to proportion of respondents choosing the most favorable option).

**Table 2:** Satisfaction with health care in the adult population of the Federation of Bosnia and Herzegovina during the COVID-19 pandemic by gender.

Question	Gender of Respondents						*p*-Value
	Females		Males		Total		
	*Mean (CI 95%)*	*CE (%)*	*Mean (CI 95%)*	*CE (%)*	*Mean (CI 95%)*	*CE (%)*	
Do you think that the family doctor spends sufficient time with you?	3 (2.8–3.1)	22%	2.9 (2.8–3)	16%	2.9 (2.8–3)	19%	0.431
Does the doctor show any interest in your problem?	3.2 (3.1–3.3)	24%	3.1 (3–3.3)	21%	3.2 (3.1–3.3)	22%	0.551
Do you feel better when you tell the doctor about your problem?	3.5 (3.4–3.7)	33%	3.3 (3.2–3.5)	27%	3.4 (3.3–3.5)	30%	0.04
Does the doctor involve you in making a decision about your treatment?	3.3 (3.2–3.5)	25%	3.4 (3.2–3.5)	24%	3.3 (3.2–3.4)	25%	0.793
Does the doctor listen to you carefully while you are presenting your problems?	3.3 (3.2–3.5)	28%	3.3 (3.2–3.5)	26%	3.3 (3.2–3.4)	27%	0.905
Does the doctor provide you with all information about your diseases?	3.3 (3.2–3.5)	26%	3.3 (3.2–3.4)	24%	3.3 (3.2–3.4)	25%	0.959
Does the doctor try hard to relieve your symptoms as soon as possible?	3.4 (3.3–3.6)	27%	3.4 (3.2–3.5)	25%	3.4 (3.3–3.5)	26%	0.503
Does the doctor help you feel better and return to your everyday work?	3.4 (3.2–3.5)	26%	3.4 (3.2–3.5)	23%	3.4 (3.2–3.5)	24%	0.974
Does the doctor perform a physical examination?	3.4 (3.3–3.6)	28%	3.6 (3.4–3.7)	30%	3.5 (3.4–3.6)	29%	0.271
Does the doctor perform a detailed physical examination?	3.2 (3–3.3)	21%	3.3 (3.1–3.4)	22%	3.2 (3.1–3.3)	22%	0.268
Does the doctor work on the prevention of various diseases?	2.9 (2.8–3)	17%	2.9 (3.8–3.1)	18%	2.9 (2.8–3)	17%	0.729
Does the doctor explain why you need to undergo additional tests and analyses?	3.2 (3.1–3.4)	24%	3.3 (3.1–3.4)	23%	3.3 (3.2–3.3)	23%	0.532
Does the doctor provide an explanation about your symptoms and disease?	3.3 (3.1–3.4)	25%	3.3 (3.2–3.4)	23%	3.3 (3.2–3.4)	24%	0.631
Does the doctor help with your emotional problems related to your health condition?	2.9 (2.8–3.1)	18%	2.9 (2.8–3.1)	17%	2.9 (2.8–3)	18%	0.931
Does the doctor explain to you why it is important to comply with his/her instructions?	3.2 (3.1–3.3)	22%	3.3 (3.1–3.4)	21%	3.2 (3.1–3.3)	22%	0.577
Does the doctor explain what he/she is doing during the examination?	3.1 (3–3.3)	22%	3.3 (3.1–3.4)	23%	3.2 (3.1–3.3)	22%	0.261
Does the doctor explain what you can expect at a specialist examination in-hospital?	2.9 (2.8–3.1)	17%	3 (2.9–3.2)	18%	3 (2.9–3.1)	17%	0.208
Are you assisted by other medical staff (nurse at the clinic)?	3.3 (3.1–3.4)	19%	3.4 (3.3–3.6)	24%	3.4 (3.3–3.4)	21%	0.079
Can you make an appointment with the doctor?	3.6 (3.4–3.7)	31%	3.5 (3.4–3.7)	32%	3.6 (3.5–3.7)	32%	0.667
Is it easy to make a phone call to the doctor?	2.9 (2.7–3)	20%	2.9 (2.8–3.1)	20%	2.9 (2.8–3)	20%	0.518
Can you seek advice from the doctor by phone?	2.9 (2.7–3)	20%	3 (2.8–3.1)	22%	3 (2.8–3)	21%	0.428
Do you wait long in the waiting room?	3.3 (3.1–3.4)	24%	3.4 (3.2–3.5)	26%	3.3 (3.2–3.4)	25%	0.271
Does the doctor respond fast in an emergency situation?	3.5 (3.4–3.6)	29%	3.4 (3.3–3.6)	29%	3.5 (3.4–3.6)	29%	0.396
Total mean score	3.2 (3.1–3.3)	24%	3.2 (3.1–3.4)	23%	3.2 (3.2–3.3)	23%	0.745

CI: confidence interval; CE: ceiling effect (refers to proportion of respondents choosing the most favorable option).

**Table 3:** Satisfaction with health care in the adult population of the Federation of Bosnia and Herzegovina during the COVID-19 pandemic by presence/absence of chronic noncommunicable disease.

Question	Chronic Disease						
	No		Yes		Total		*p*-Value
	*Mean (CI 95%)*	*CE (%)*	*Mean (CI 95%)*	*CE (%)*	*Mean (CI 95%)*	*CE (%)*	
Do you think that the family doctor spends sufficient time with you?	3 (2.8–3.1)	19%	3.1 (2.9–3.3)	25%	2.9 (2.8–3)	20%	0.321
Does the doctor show any interest in your problem?	3.2 (3.1–3.3)	22%	3.3 (3.1–3.5)	28%	3.2 (3.1–3.3)	24%	0.412
Do you feel better when you tell the doctor about your problem?	3.4 (3.2–3.5)	28%	3.7 (3.5–3.9)	39%	3.4 (3.3–3.5)	31%	<0.01
Does the doctor involve you in making a decision about your treatment?	3.4 (3.3–3.5)	24%	3.4 (3.2–3.6)	32%	3.3 (3.2–3.4)	26%	0.704
Does the doctor listen to you carefully while you are presenting your problems?	3.4 (3.3–3.5)	26%	3.5 (3.3–3.7)	34%	3.3 (3.2–3.4)	28%	0.378
Does the doctor provide you with all information about your diseases?	3.3 (3.2–3.5)	24%	3.5 (3.3–3.7)	32%	3.3 (3.2–3.4)	26%	0.235
Does the doctor try hard to relieve your symptoms as soon as possible?	3.4 (3.3–3.6)	27%	3.5 (3.3–3.7)	31%	3.4 (3.3–3.5)	28%	0.875
Does the doctor help you feel better and return to your everyday work?	3.4 (3.3–3.5)	24%	3.5 (3.2–3.7)	31%	3.4 (3.2–3.5)	26%	0.634
Does the doctor perform a physical examination?	3.6 (3.5–3.7)	31%	3.3 (3.1–3.6)	31%	3.5 (3.4–3.6)	31%	0.028
Does the doctor perform a detailed physical examination?	3.3 (3.2–3.4)	23%	3.2 (3–3.4)	24%	3.2 (3.1–3.3)	23%	0.332
Does the doctor work on the prevention of various diseases?	3 (2.9–3.1)	18%	2.8 (2.6–3)	21%	2.9 (2.8–3)	19%	0.104
Does the doctor explain why you need to undergo additional tests and analyses?	3.3 (3.2–3.4)	25%	3.2 (3–3.4)	25%	3.3 (3.2–3.3)	25%	0.357
Does the doctor provide an explanation about your symptoms and disease?	3.3 (3.2–3.5)	25%	3.3 (3–3.5)	28%	3.3 (3.2–3.4)	26%	0.503
Does the doctor help with your emotional problems related to your health condition?	3 (2.9–3.1)	18%	2.9 (2.7–3.2)	21%	2.9 (2.8–3)	19%	0.753
Does the doctor explain to you why it is important to comply with his/her instructions?	3.3 (3.2–3.4)	22%	3.3 (3–3.4)	25%	3.2 (3.1–3.3)	23%	0.831
Does the doctor explain what he/she is doing during the examination?	3.3 (3.2–3.4)	23%	3.2 (2.9–3.4)	26%	3.2 (3.1–3.3)	24%	0.284
Does the doctor explain what you can expect at a specialist examination in-hospital?	3.1 (3–3.2)	19%	3 (2.8–3.2)	18%	3 (2.9–3.1)	19%	0.368
Are you assisted by other medical staff (nurse at the clinic)?	3.4 (3.3–3.5)	21%	3.5 (3.3–3.7)	28%	3.4 (3.3–3.4)	23%	0.548
Can you make an appointment with the doctor?	3.6 (3.4–3.7)	30%	3.7 (3.5–3.8)	42%	3.6 (3.5–3.7)	33%	0.365
Is it easy to make a phone call to the doctor?	3 (2.8–3.1)	19%	2.9 (2.6–3.1)	26%	2.9 (2.8–3)	21%	0.508
Can you seek advice from the doctor by phone?	3 (2.9–3.1)	20%	2.8 (2.6–3.1)	25%	3 (2.8–3)	22%	0.257
Do you wait long in the waiting room?	3.3 (3.2–3.5)	23%	3.2 (3–3.4)	26%	3.3 (3.2–3.4)	24%	0.351
Does the doctor respond fast in an emergency situation?	3.5 (3.4–3.6)	28%	3.7 (3.5–3.9)	39%	3.5 (3.4–3.6)	31%	0.069
**Total mean score**	**3.3 (3.1–3.4)**	**23%**	**3.3 (3.1–3.4)**	**29%**	**3.2 (3.2–3.3)**	**25%**	**1**

CI: confidence interval; CE: ceiling effect (refers to proportion of respondents choosing the most favorable option).

**Table 4:** Satisfaction with health care in the adult population of the Federation of Bosnia and Herzegovina during the COVID-19 pandemic by residence.

Question	Residence						
	Rural		Urban		Total		*p*-Value
	*Mean (CI 95%)*	*CE (%)*	*Mean (CI 95%)*	*CE (%)*	*Mean (CI 95%)*	*CE (%)*	
Do you think that the family doctor spends sufficient time with you?	2.9 (2.7–3.1)	18%	3 (2.8–3.1)	20%	2.9 (2.8–3)	19%	0.539
Does the doctor show any interest in your problem?	3.2 (3–3.3)	21%	3.2 (3.1–3.3)	23%	3.2 (3.1–3.3)	22%	0.879
Do you feel better when you tell the doctor about your problem?	3.4 (3.3–3.6)	28%	3.4 (2.3–3.5)	31%	3.4 (3.3–3.5)	30%	0.894
Does the doctor involve you in making a decision about your treatment?	3.4 (3.2–3.5)	24%	3.3 (3.2–3.4)	25%	3.3 (3.2–3.4)	25%	0.543
Does the doctor listen to you carefully while you are presenting your problems?	3.4 (3.2–3.5)	26%	3.3 (3.2–3.5)	27%	3.3 (3.2–3.4)	27%	0.822
Does the doctor provide you with all information about your diseases?	3.3 (3.2–3.5)	24%	3.3 (3.2–3.4)	25%	3.3 (3.2–3.4)	25%	0.941
Does the doctor try hard to relieve your symptoms as soon as possible?	3.4 (3.3–3.6)	24%	3.4 (3.3–3.5)	27%	3.4 (3.3–3.5)	26%	0.796
Does the doctor help you feel better and return to your everyday work?	3.4 (3.2–3.5)	22%	3.4 (3.2–3.5)	26%	3.4 (3.2–3.5)	24%	0.923
Does the doctor perform a physical examination?	3.6 (3.4–3.7)	28%	3.5 (3.3–3.6)	30%	3.5 (3.4–3.6)	29%	0.441
Does the doctor perform a detailed physical examination?	3.2 (3.1–3.4)	19%	3.2 (3.1–3.3)	24%	3.2 (3.1–3.3)	22%	0.997
Does the doctor work on the prevention of various diseases?	2.9 (2.8–3.1)	17%	2.9 (2.8–3)	18%	2.9 (2.8–3)	17%	0.846
Does the doctor explain why you need to undergo additional tests and analyses?	3.3 (3.1–3.4)	22%	3.2 (3.1–3.4)	24%	3.3 (3.2–3.3)	23%	0.879
Does the doctor provide an explanation about your symptoms and disease?	3.3 (3.1–3.4)	21%	3.3 (3.2–3.4)	25%	3.3 (3.2–3.4)	24%	0.753
Does the doctor help with your emotional problems related to your health condition?	2.9 (2.7–3.1)	16%	2.9 (3.8–3.1)	18%	2.9 (2.8–3)	18%	0.768
Does the doctor explain to you why it is important to comply with his/her instructions?	3.3 (3.1–3.4)	20%	3.2 (3.1–3.3)	23%	3.2 (3.1–3.3)	22%	0.685
Does the doctor explain what he/she is doing during the examination?	3.2 (3.1–3.4)	20%	3.2 (3.1–3.3)	24%	3.2 (3.1–3.3)	22%	0.759
Does the doctor explain what you can expect at a specialist examination in-hospital?	3 (2.9–3.2)	16%	3 (2.8–3.1)	18%	3 (2.9–3.1)	17%	0.6
Are you assisted by other medical staff (nurse at the clinic)?	3.4 (3.3–3.6)	22%	3.3 (3.2–3.4)	21%	3.4 (3.3–3.4)	21%	0.355
Can you make an appointment with the doctor?	3.6 (3.4–3.7)	29%	3.5 (3.4–3.7)	33%	3.6 (3.5–3.7)	32%	0.82
Is it easy to make a phone call to the doctor?	2.9 (2.8–3.1)	19%	2.9 (2.8–3)	20%	2.9 (2.8–3)	20%	0.723
Can you seek advice from the doctor by phone?	3 (2.9–3.2)	21%	2.9 (2.8–3)	21%	3 (2.8–3)	21%	0.495
Do you wait long in the waiting room?	3.3 (3.1–3.4)	20%	3.3 (3.2–3.5)	28%	3.3 (3.2–3.4)	25%	0.468
Does the doctor respond fast in an emergency situation?	3.5 (3.4–3.7)	30%	3.5 (3.3–3.6)	29%	3.5 (3.4–3.6)	29%	0.571
**Total mean score**	**3.2 (3.1–3.4)**	**22%**	**3.2 (3.1–3.3)**	**24%**	**3.2 (3.2–3.3)**	**23%**	**0.81**

CI: confidence interval; CE: ceiling effect (refers to proportion of respondents choosing the most favorable option).

**Table 5:** Satisfaction with health care in the adult population of the Federation of Bosnia and Herzegovina compared with the region of Zenica-Doboj during the COVID-19 pandemic by age group.

Question	FB&H	Zenica-Doboj Region	*p*-Value
Do you think that the family doctor spends sufficient time with you?	2.9 (2.8–3)	3 (2.8–3.3)	0.371
Does the doctor show any interest in your problem?	3.1 (3.0–3.2)	3.3 (3.1–3.5)	0.117
Do you feel better when you tell the doctor about your problem?	3.4 (3.3–3.5)	3.4 (3.2–3.6)	0.812
Does the doctor involve you in making a decision about your treatment?	3.3 (3.2–3.4)	3.4 (3.2–3.6)	0.311
Does the doctor listen to you carefully while you are presenting your problems?	3.3 (3.2–3.4)	3.4 (3.2–3.6)	0.409
Does the doctor provide you with all information about your diseases?	3.2 (3.1–3.3)	3.3 (3.1–3.5)	0.954
Does the doctor try hard to relieve your symptoms as soon as possible?	3.4 (3.3–3.5)	3.4 (3.2–3.6)	0.923
Does the doctor help you feel better and return to your everyday work?	3.5 (3.4–3.6)	3.5 (3.3–3.7)	0.173
Does the doctor perform a physical examination?	3.5 (3.4–3.6)	3.6 (3.4–3.8)	0.166
Does the doctor perform a detailed physical examination?	3.2 (3.1–3.3)	3.3 (3.1–3.5)	0.286
Does the doctor work on the prevention of various diseases?	2.9 (2.8–3)	2.9 (2.7–3.1)	0.695
Does the doctor explain why you need to undergo additional tests and analyses?	3.3 (3.2–3.3)	3.3 (3–3.5)	1
Does the doctor provide an explanation about your symptoms and disease?	3.3 (3.2–3.4)	3.3 (3–3.5)	0.936
Does the doctor help with your emotional problems related to your health condition?	3.0 (2.9–3.1)	3 (2.7–3.2)	0.729
Does the doctor explain to you why it is important to comply with his/her instructions?	3.2 (3.1–3.3)	3.2 (3–3.4)	0.941
Does the doctor explain what he/she is doing during the examination?	3.2 (3.1–3.3)	3.2 (3–3.4)	0.766
Does the doctor explain what you can expect at a specialist examination in-hospital?	3 (2.9–3.1)	3 (2.8–3.2)	0.713
Are you assisted by other medical staff (nurse at the clinic)?	3.4 (3.3–3.4)	3.3 (3.1–3.5)	0.723
Can you make an appointment with the doctor?	3.6 (3.5–3.7)	3.9 (3.7–4.1)	<0.01
Is it easy to make a phone call to the doctor?	2.8 (2.7–2.9)	3.3 (3.1–3.5)	<0.001
Can you seek advice from the doctor by phone?	2.9 (2.8–3)	3.1 (2.9–3.3)	0.118
Do you wait long in the waiting room?	3.3 (3.2–3.4)	3.2 (3–3.4)	0.491
Does the doctor respond fast in an emergency situation?	3.5 (3.4–3.6)	3.6 (3.4–3.8)	0.324
**Total mean score**	**3.3 (3.1–3.5)**	**3.2 (3.1–3.3)**	**0.45**

For these comparisons, respondents from the Zenica-Doboj region were excluded from the “FB&H” group; means with 95% confidence intervals are presented.

## Data Availability

Data are available from the lead author upon reasonable request.
